# Climate-human interaction associated with southeast Australian megafauna extinction patterns

**DOI:** 10.1038/s41467-019-13277-0

**Published:** 2019-11-22

**Authors:** Frédérik Saltré, Joël Chadoeuf, Katharina J. Peters, Matthew C. McDowell, Tobias Friedrich, Axel Timmermann, Sean Ulm, Corey J. A. Bradshaw

**Affiliations:** 1 Global Ecology, College of Science and Engineering and ARC Centre of Excellence for Australian Biodiversity and Heritage, Flinders University, GPO Box 2100, Adelaide, SA 5001 Australia; 2 UR 1052, French National Institute for Agricultural Research (INRA), Montfavet, France; 3 Dynamics of Eco-Evolutionary Pattern and ARC Centre of Excellence for Australian Biodiversity and Heritage, University of Tasmania, Tasmania, 7001 Australia; 4 University of Hawai’i at Mānoa, Honolulu, HI USA; 5 Center for Climate Physics, Institute for Basic Science, Busan, 46241 Korea; 6 Pusan National University, Busan, 46241 Korea; 7 ARC Centre of Excellence for Australian Biodiversity and Heritage, College of Arts, Society and Education, James Cook University, PO Box 6811, Cairns, QLD 4870 Australia

**Keywords:** Biodiversity, Macroecology, Palaeoecology, Archaeology

## Abstract

The mechanisms leading to megafauna (>44 kg) extinctions in Late Pleistocene (126,000—12,000 years ago) Australia are highly contested because standard chronological analyses rely on scarce data of varying quality and ignore spatial complexity. Relevant archaeological and palaeontological records are most often also biased by differential preservation resulting in under-representated older events. Chronological analyses have attributed megafaunal extinctions to climate change, humans, or a combination of the two, but rarely consider spatial variation in extinction patterns, initial human appearance trajectories, and palaeoclimate change together. Here we develop a statistical approach to infer spatio-temporal trajectories of megafauna extirpations (local extinctions) and initial human appearance in south-eastern Australia. We identify a combined climate-human effect on regional extirpation patterns suggesting that small, mobile Aboriginal populations potentially needed access to drinkable water to survive arid ecosystems, but were simultaneously constrained by climate-dependent net landscape primary productivity. Thus, the co-drivers of megafauna extirpations were themselves constrained by the spatial distribution of climate-dependent water sources.

## Abstract (web summary)

Whether Australia’s Pleistocene megafauna extinctions were caused by climate change, humans, or both is debated. Here, the authors infer the spatio-temporal trajectories of regional extinctions and find that water availability mediates the relationship among climate, human migration and megafauna extinctions.

## Introduction

The Late Pleistocene megafauna (usually defined as animals >44 kg) extinction wave was a rapid, large-scale ecological phenomenon of unprecedented magnitude in the Quaternary^1^. However, the causes and consequences of these extinctions are still unresolved^2^. Many conceptual models have been developed to explain the causes of megafauna extinctions, which can be classified into the following three themes: (i) climatic changes increasingly restricted suitable habitats for megafauna species^3^; (ii) the spread of humans—a new and efficient top predator—from Africa into other continents negatively affected megafauna either by exploiting species’ naivety, or profoundly modifying their ecosystems^4,5^; or (iii) a possible combination of human exploitation of populations already compromised by climate-driven environmental changes (or vice versa)^6^.

Most scientific contributions to the relative weight of these three themes rely exclusively on chronological analyses and ignore spatial patterns entirely—the general approach is to compare the timing of both megafauna extinctions and the initial arrival of humans (associated with the age of the last and first palaeontological and archaeological records, respectively) to the reconstruction of contemporaneous, relative climate time series^1,3,7–10^. Such analyses are challenging because robust data are rare, and inferences are often biased because older events are under-represented due to differential preservation of evidence over time^11^, and both the oldest and youngest ages of a palaeo time series are inaccurate reflections of an event^12^. This phenomenon is exacerbated in Australia where the average density of dated archaeological sites is <1 per 4000 km^2^ and only 1 per 10000 km^2^ in lesser-studied regions^13^. Statistical methods that attempt to overcome these chronological biases (reviewed in ref. ^14^) are either not spatially explicit and disregard spatial variation in extinction patterns, initial human appearance trajectories, and palaeoclimate change^7,15^, or they generate new spatial biases via arbitrary geographic binning^8–10^ or spatially continuous estimation^16^ that do not account for the uncertainty arising from sampling and taphonomic biases^17^, inherent dating errors^18^, or spatial biases generated when interpolating a linear chronology from unevenly spaced age estimates.

We overcome these methodological limitations by developing and applying a statistical approach to the available data describing megafauna extirpations and patterns of human appearance in south-eastern Australia over the last 120,000 years to test three explicit hypotheses: (1) megafauna extirpations followed changing climate conditions, (2) megafauna extirpations followed the arrival of Aboriginal populations in the region, or (3) megafauna extirpations followed a combination of both climate change and human arrival. To test these hypotheses, we generate maps of continuous regional timings of megafauna extirpations and initial human appearance, and apply generalised least-squares models to explore their relative roles in explaining both the regional timing and directions of the spatial gradients (bearings) of megafauna extirpations across south-eastern Australia. We also account for possible temporal lags, because extinctions could have been driven by earlier climatic variation^7^ (see Methods). We show that (i) >80% of south-eastern Australia had a period of human-megafauna coexistence lasting from 1000 to >15,000 years, and (ii) the pattern of megafauna extirpation in these areas is best explained by an additive effect of the patterns of human spread and freshwater availability across the region. These findings suggest that only by adding spatial complexity into standard chronological analyses can we explicitly identify both humans and climate change as the most likely drivers of these extinctions.

## Results

### Regional patterns of megafauna extirpation and human spread

**Fig. 1:**
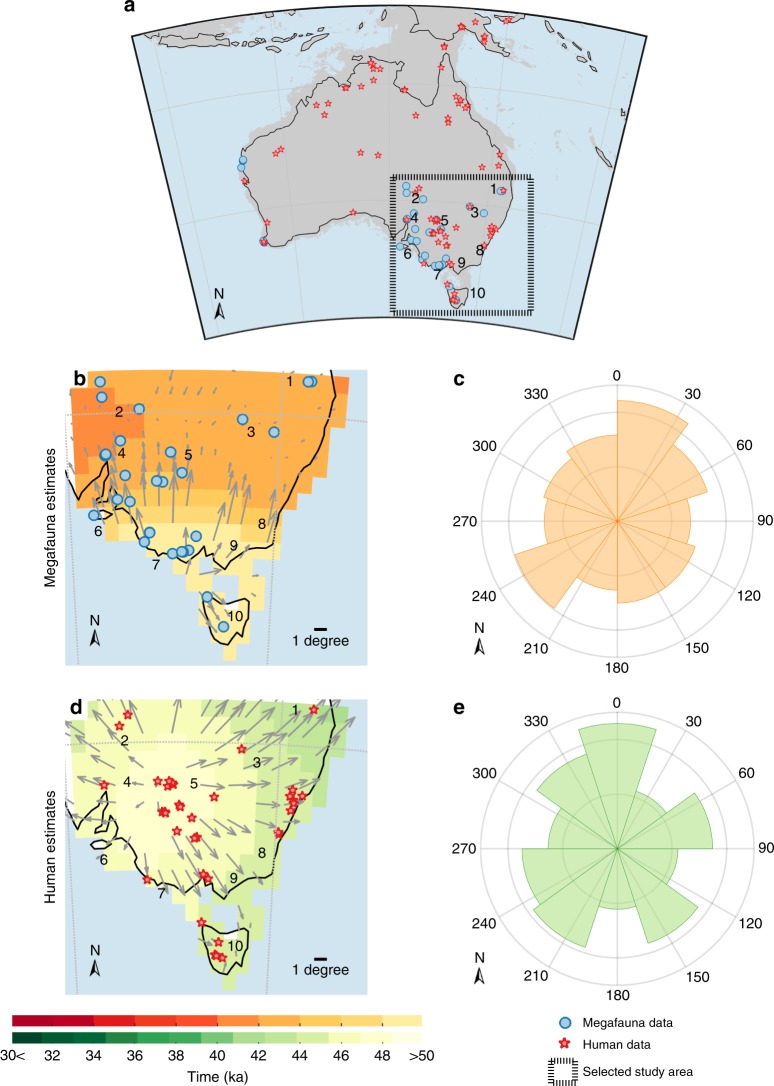
Spatial pattern of timing of megafauna extirpation and first human arrival. **a** Regional estimates in south-eastern Australia of the **b** timing and **c** bearing of megafauna extirpation and **d** timing and **e** bearing of initial human arrival, from high quality-rated ages of megafauna fossils (blue circles) and archaeological specimens (red stars) calculated at a spatial resolution of 1 × 1°. Grey arrows represent the spatial gradient of megafauna extirpation and human appearance from the oldest to the youngest point estimates. The angle-histogram polar plots in **c**, **e** show the distribution of bearings (calculated in degrees) of the spatial gradient of timings of **c** megafauna extirpation and **e** initial human arrival. These angle-histogram polar plots are normalised so that the height of each bar represents the number of grid cells in the bin (specific bearing) divided by the total number of observations multiplied by the width of the bin. The area of each bar is the number of grid cells, and the sum of the bar areas = 1. The spatial component is not included in these plots, i.e., the centre of these angle-histogram polar plots do not relate to any particular geographic location on the map. Each region is named as follows: Sandy Hollow Creek (1), Lake Eyre (2), Eastern Great Dividing Ranges (3), Port Augusta (4), Willandra Lakes (5), Kangaroo Island (6), southeast coastal plains (7), Sydney Basin (8), southern coast of mainland Australia (9), and Tasmania (10).

We first identified the regional locations where and periods during which humans had putatively coexisted with megafauna species within south-eastern Australia by comparing their respective regional chronologies (i.e., extirpation and initial arrival) and their spatial bearings. To estimate an accurate regional chronology for these patterns, we applied a spatio-temporal statistical method (see details in Methods and Supplementary Table 1) to high-quality (i.e., suitable dates; see quality-rating approach in Methods) megafauna fossil records and to the archaeology of initial human appearance of Sahul (Fig. 1a, Supplementary Table 1 and Supplementary Data 1). Our method returns for each 1 × 1° terrestrial grid cell in south-eastern Australia a mean estimate of both a timing of megafauna extirpation and initial human arrival along with their respective standard deviations (see details in Methods and Supplementary Note 1).

The regional pattern of megafauna extirpations started around 48 ka (1 ka = 1000 years ago) in the southeast coastal plains (location 7; Fig. 1b) and the southern coast of mainland Australia (location 9). Megafauna extirpations mostly radiated (i) 0–60° north from location 7 (Fig. 1c) to reach locations 5 and 8 between ~46 and 42 ka, via location 9 (Fig. 1a), (ii) 110 to 180° south from locations 7 and 9 to reach location 10 (Tasmania) between ~46 ka (Fig. 1b), and continued both (iii) 210 to 240° south from location 1 and (iv) 330 to 0 north from location 6 (Kangaroo Island) to reach locations 2 (Lake Eyre) and 4 by ~42 and 40 ka. In contrast, the pattern of the peopling of the region started >50 ka from location 5 (Fig. 1d), then mostly radiated northward (310° to the northwest; Fig. 1e) to location 2 by ~44 ka via location 4 (Port Augusta), and location 1 by ~41 ka (Fig. 1d) via location 3 (Darling Downs, ~44 ka). Humans reached location 10 (Tasmania) by ~42 ka from the northwest via location 7 (~44 ka) and later (~42 ka) from the northeast via the southern coast of mainland Australia at location 9.

### Drivers of coexistence/non-coexistence areas

**Fig. 2:**
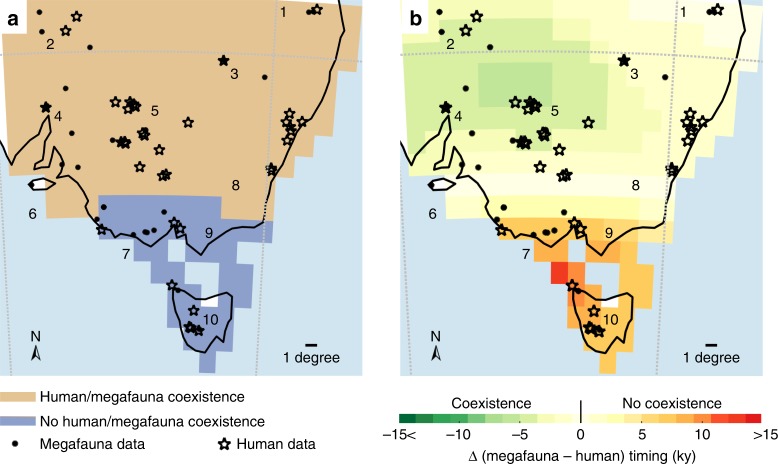
Spatial comparison of estimated timing of megafauna extirpation and initial human arrival at a spatial resolution of 1 × 1°. **a** Areas of coexistence (brown grid cells) and non-coexistence (blue grid cells) between humans and megafauna. For each grid cell, we indicated whether the timing of human arrival preceded (i.e., coexistence = humans arrived before megafauna were extirpated) or followed (i.e., no coexistence = humans arrived after megafauna were extirpated) megafauna extirpation. We accounted for confidence intervals around estimated timings so that ‘coexistence’ indicates when the lower confidence limit of the timing of initial human arrival is older than the lower confidence interval limit of megafauna extirpation. We discuss the sensitivity of the size of these areas to the approach we used to take into account these confidence intervals in Supplementary Note 1. **b** Duration of the window of coexistence/non-coexistence between humans and megafauna. For each grid cell, we subtracted from the estimated timing of extirpation the timing of initial human arrival. Negative values (from light yellow to dark green) indicate how long humans coexisted with megafauna (i.e., the darker the green, the longer they coexisted), whereas positive values (from light yellow to dark red) indicate how long megafauna had been gone prior to the arrival of the first humans (i.e., the darker the red, the longer megafauna had been absent).

The regional chronology analysis indicated that 81.1% (ranging from 69 to 84%, depending on the criteria used; see Supplementary Note 2 for details) of the 1 × 1° grid cells (Fig. 2a) had a period of human-megafauna coexistence lasting between 1000 (east of location 3, and between locations 6 and 8; Fig. 2b) and 8000 years (between locations 2 and 5; Fig. 2a). In these areas of temporal overlap, we built 60 generalised least-squares models to explain variation in the timing of megafauna extirpations (Ext_*t*_) (Table 1) as a function of all possible combinations of mean annual temperature anomaly, mean annual precipitation anomaly, mean annual water availability anomaly, mean annual net primary production anomaly, mean annual percentage of desert anomaly, and the timing of initial human arrival (see model description in Methods). We obtained the corresponding climate data from a transient LOVECLIM^19^ Earth-system model experiment, which uses time-varying orbital forcings, ice-sheet volume, and greenhouse gases as a forcing covering the past 784,000 years. We calculated the anomalies relative to the period from 50 to 30 ka (see details in Methods).

**Table 1:** Performance values for the top-ranked generalised least-squares models.

Model	Area	Lag(ka)	k	DE(%)	AIC_c_	wAIC_c_	LogLik
Ext_*t*_ ~ *P*_*a*_ + EminP_*a*_ + DF_*a*_ + (*P*_*a*_ × EminP_*a*_) + (DF_*a*_ × EminP_*a*_)	n/coexist	0	8	99.4	634.4	0.6	−306.7
Ext_*t*_ ~ *P*_*a*_ + EminP_*a*_ + DF_*a*_	n/coexist	1	6	99.9	645.8	0.4	−315.5
Ext_*t*_ ~ *P*_*a*_ + EminP_*a*_ + DF_*a*_	n/coexist	2	6	48.8	643.7	0.3	−314.5
Ext_*t*_ ~ *P*_*a*_ + EminP_*a*_ + DF_*a*_	n/coexist	3	6	10.1	644.8	0	−315.1
Ext_*t*_ ~ none	n/coexist	4	–	–	–	–	–
Ext_*t*_ ~ none	n/coexist	5	–	–	–	–	–
Ext_*t*_ ~ none	coexist	0,1,2,3,4,5	–	–	–	–	–
Ext_*b*_ ~ NPP_*b*_	n/coexist	0	4	98.8	444.9	0.3	−217.8
Ext_*b*_ ~ *T*_*b*_ + *P*_*b*_	n/coexist	1	6	74.7	1875.9	0.6	−931.7
Ext_*b*_ ~ *T*_*b*_ + NPP_*b*_ + (*T*_*b*_ × *P*_*b*_)	n/coexist	2	7	73.1	1865.5	0.9	−925.4
Ext_*b*_ ~ *T*_*b*_ + NPP_*b*_ + (*T*_*b*_ × *P*_*b*_)	n/coexist	3	7	70.4	1866.9	0.8	−926.1
Ext_*b*_ ~ *T*_*b*_ + NPP_*b*_	n/coexist	4	5	66.6	1873.1	0.6	−931.3
Ext_*b*_ ~ *T*_*b*_ + NPP_*b*_	n/coexist	5	5	68.9	1876.9	0.9	−933.3
Ext_*b*_ ~ *H*_*b*_ + *P*_*b*_ + EminP_b_	coexist	0	6	81.2	1886.9	0.4	−937.2
Ext_*b*_ ~ *H*_*b*_ + NPP_*b*_	coexist	1	5	99.9	422.7	0.8	−205.4
Ext_*b*_ ~ *H*_*b*_ + NPP_*b*_	coexist	2	5	99.9	422.9	0.7	−205.5
Ext_*b*_ ~ *H*_*b*_ + NPP_*b*_	coexist	3	5	99.9	423.1	0.7	−205.6
Ext_*b*_ ~ *H*_*b*_ + NPP_*b*_	coexist	4	5	99.9	424.2	0.7	−206.2
Ext_*b*_ ~ *H*_*b*_ + NPP_*b*_	coexist	5	5	99.9	424.6	0.7	−206.4

These top-ranked generalised least-squares models contain climate (*T* = mean annual temperature, *P* = mean annual precipitation, EminP = mean annual freshwater availability, NPP = mean annual net primary production and DF = fraction of desert within the grid cell), and human predictors (*H*) to describe (i) the timing of megafauna extirpation (Ext_*t*_) in human-megafauna non-coexistence areas (n/coexist) and (ii) in human-megafauna coexistence areas (coexist), (iii) the bearing of timing of megafauna extirpation (Ext_*b*_) in human-megafauna non-coexistence areas (n/coexist) and (iv) in areas with coexistence (coexist). For each of these four scenarios we included five temporal lags (Lag) between the climate from 0 to 5 ka (at a 1 ka-year time step, with ka = 1000 years) for the period earlier than the estimated timing of megafauna extirpation in each grid cell. Predictor variables subscripted *a* (*P*_*a*_, EminP_*a*_, DF_*a*_) indicate that we used the mean annual anomaly relative to the period 50–30 ka for these variables, whereas predictor variables subscripted *b* (*T*_*b*,_ *P*_*b*_, NPP_*b*_, EminP_*b*_, *H*_*b*_) indicate that we used the directional bearing. Shown are the number of parameters (k), metric of the model’s structural goodness of fit (%DE), minimised negative log-likelihood (LogLik), weight scaled to a sum of 1 (wAIC_*c*_) and the Akaike’s information criterion corrected for small sample sizes (AIC_*c*_) for the highest-ranking model. See details of the full list of generalised least-squares models describing all the combinations among variable predictors in Supplementary Data 2

**Fig. 3:**
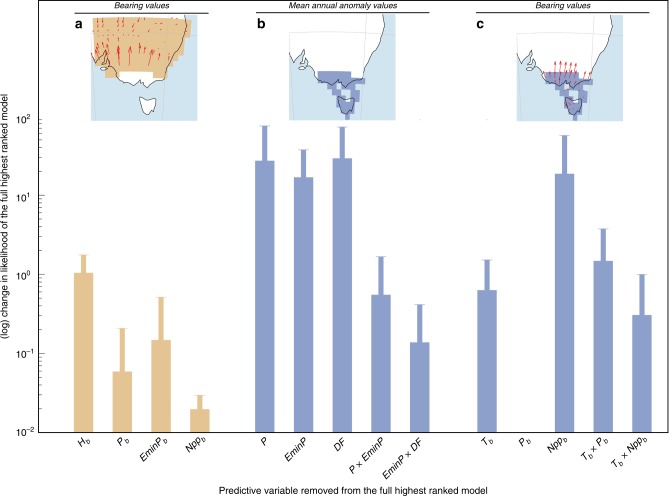
Most likely drivers of megafauna extirpation timings and patterns. Relative importance of predictor variables for the top-ranked generalised least-squares model assuming a Gaussian spatial autocorrelation structure best decribing **a** the spatial gradient (bearings) of megafauna extirpation (Ext_*b*_) in human-megafauna coexistence areas, **b** the timing of megafauna extirpation (Ext_*t*_) in human-megafauna non-coexistence areas and **c** the bearings of megafauna extirpation (Ext_*b*_) in human-megafauna non-coexistence areas. Human-megafauna coexistence and non-coexistence areas are described in Fig. 2a and in the top panel of each barplot in Fig. 3 along with red arrows indicating the bearing of megafauna extinction gradients. The relative importance of each predictor variable is calculated as the change in the full model likelihood when one of its predictor variables is removed. Climate predictors were from LOVECLIM^19,39^ for the time period in each grid cell corresponding to the estimated timing of megafauna extirpation and its confidence interval. Predictor variables of the model describing Ext_*t*_ are mean annual temperature anomaly (*T*), mean annual precipitation anomaly (*P*), mean annual freshwater availability anomaly (EminP), mean annual net primary production anomaly (NPP), and the fraction of desert anomaly within the grid cell (DF). Climate anomalies are calculated relative to the 50–30 ka mean time period (see Methods). Predictor variables subscripted *b* (*T*_*b*_, EminP_*b*_) indicate that we used the directional bearing of these climate variables, including the directional vectors for the timing of initial human arrival (*H*_*b*_), to build the model describing the spatial pattern bearing of megafauna extirpation (Ext_*b*_). For clarity, we did not present the results describing Ext_*t*_ for the areas with human and megafauna coexistence because we did not have any relevant model (i.e., percentage of variance explained by the models ~ 0%) in those areas. For each variable, error bars represent the standard deviation of the relative importance of predictor variables for the top-ranked generalised least-squares model accounting for the temporal lag by regressing extirpation against climate from 0 to 5000 years (at a 1000-year time step = 5 temporal-lag scenarios, i.e., 6 values of relative importance in total) per grid cell. Source data are provided as a Source Data file.

None of the generalised least-squares models we tested explained any variation in the timing alone of megafauna extirpation in areas of human-megafauna coexistence (i.e., ~0% of variance explained). However, the top-ranked model explained variation in the spatial pattern of timing of extirpation (bearing = Ext_*b*_; Table 1), such as the bearing of the timing of human arrival, the bearing of mean annual precipitation, and water availability (>81.2% of variance explained; Table 1). Both the bearing of the timing of human arrival and the bearing of water availability had the strongest effects on the change in estimated likelihood for this model (Fig. 3a). This indicates that these variables are the main contributors to the goodness of fit (i.e., how well this model fits the data of timing of megafauna extirpations), even accounting for up to 5000 years of possible temporal lags in climate variation.

Regions such as locations 7, 9 and 10 (19% of the grid cells ranging from 16 to 31%; Fig. 2a, and Supplementary Figs. 4 and 5) indicated no period of human-megafauna coexistence because the first humans arrived there between 5000 (e.g., locations 5 and 9) and >9000 years (location 10; Fig. 2b) after megafauna species went locally extinct. A generalised least-squares model built on mean annual precipitation anomaly (*P*), mean annual water availability anomaly (EminP), the percentage of desert fraction (DF), and their respective interactions (i.e., *P* × EminP and EminP × DF) as predictive variables provided the highest support to describe the timing of extirpation in these areas (>99.4% of the variance explained and the lowest AIC_*c*_; Table 1). These three predictive variables provide the highest contribution in this model (Fig. 3b), even accounting for up to 5000 years of possible temporal lags in climate variation. The coefficients of the highest-ranked generalised least-squares model (Table 1) indicate that the oldest extinction events are associated with both lowest mean annual precipitation anomalies, mean annual desert anomalies (i.e, both with negative coefficients; Table 2), and mean annual freshwater availability anomalies (i.e., positive EminP means higher evapotranspiration relative to precipitation influx, which means less available freshwater; Table 2). However, the bearing of net primary productivity (NPP_*b*_) provides the highest support to the spatial pattern of timing of extirpation (bearing = Ext_*b*_; Table 1 and Fig. 3c) in these areas, while the bearing of mean annual temperature became an important explanatory variable when accounting for the temporal lag in climate. The negative NPP_*b*_ coefficient (Table 2) means that the regional pattern of megafauna extinction (Ext_*b*_) and net primary production (NPP_*b*_) shifted in an oppositive direction.

**Table 2:** Summary statistics for the top-ranked generalised least-squares models.

	Area	Variable	Coefficient		Correlation								
			value	std error	*P*_*a*_	EminP_*a*_	DF_*a*_	*P*_*a*_ × EminP_*a*_	DF_*a*_ × EminP_*a*_	NPP_*b*_	*H*_*b*_	*P*_*b*_	EminP_*b*_
Ext_*t*_ ~ *P*_*a*_ + EminP_*a*_ + DF_*a*_ + (*P*_*a*_ *×* EminP_*a*_) + (DF_*a*_ × EminP_*a*_)	n/coexist	*P*_*a*_	−4098.61	555.92	1	0.01	0.63	−0.66	−0.28				
		EminP_*a*_	378.48	784.24		1	0.29	0.42	−0.18	–	–	–	–
		DF_*a*_	−513.70	1044.20			1	−0.39	−0.69	–	–	–	–
		*P*_*a*_ × EminP_*a*_	3592.36	1340.64				1	−0.29	–	–	–	–
		DF_*a*_ × EminP_*a*_	−10519.01	3238.69					1	–	–	–	–
Ext_*b*_ ~ NPP_*b*_	n/coexist	NPP_*b*_	−0.38	0.09						1	–	–	–
Ext_*b*_ ~ *H*_*b*_ + *P*_*b*_ + EminP_*b*_	coexist	*H*_*b*_	0.34	0.09							1	0.04	0.33
		*P*_*b*_	−0.18	0.09								1	−0.18
		EminP_*b*_	−0.13	0.07									1

Coefficients estimated for each predictor variable of the top-ranked generalised least-squares model, standard error, and correlation between predictor variables to describe the timing of megafauna extirpation (Ext_*t*_) and its bearing (Ext_*b*_) in human-megafauna non-coexistence (n/coexist) and coexistence (coexist) areas. Climate variables are mean annual temperature (*T*), mean annual precipitation (*P*), mean annual freshwater availability (EminP), mean annual net primary production (NPP), and the fraction of desert within the grid cell (DF), and the human predictor is the estimated timing of initial human arrival (*H*). Predictor variables subscripted *a* (*P*_*a*_, EminP_*a*_, DF_*a*_) indicate that we used the mean annual anomaly relative to the period 50–30 ka for these variables, whereas predictor variables subscripted *b* (*P*_*b*_, NPP_*b*_, EminP_*b*_, *H*_*b*_) indicate that we used the directional bearing. For clarity, we did not present the results of the model best describing Ext_*t*_ in the areas with human and megafauna coexistence because we did not have any model with a percentage of variance explained > 0% in those areas

## Discussion

Our results support an additive effect of available freshwater and human arrival on megafauna extirpations in south-eastern Australia, at least in terms of the complex regional pattern (i.e., bearing) of change (Table 1 and Fig. 3a). The combination of climate change and human pressure has been suggested in global and continental analyses as the most plausible set of conditions explaining megafauna extinctions^8,9,15,20^. Here, the loss of suitable habitat driven by rapid climate shifts (i.e., a shift in temperature and precipitation) would have been important precursor conditions exacerbating the effects of humans on megafauna. Similar results are also supported by more regional and local palaeoecological analyses in South America concluding that megafaunal extinctions did not occur until human appearance and warming coincided^21^, with humans potentially limiting species recovery after climate-induced stress^22^.

This result in such a continuous, spatially explicit context cannot be explored by using standard chronological approaches (i.e., without integrating a spatial component—the bearing—that connects all grid cells together instead of considering them as independent entities), because none of the 60 generalised least-squares models we tested explained any variation in the timing alone of megafauna extirpation in areas of human-megafauna coexistence (Table 1). Such an interaction between the regional patterns of a climate-driven variable (available freshwater) and those of human appearance suggests that the first humans in this region migrated across Australia by exploiting palaeolakes and other sources of drinkable water connecting the drier regions in between^23^. Given that approximatively three-quarters of Australia is semi-arid or arid, it is plausible that megafauna species were attracted to the same freshwater resources as were humans^24^, thus increasing the chances of encountering humans after the latter had arrived. We contend that these water points became hotspots of interspecific competition for water resources and human hunting that affected megafauna persistence depending on the particular configuration of freshwater resources in the local landscape.

Despite this effect of human appearance on the regional pattern of megafauna extirpations (Table 1), we found no effect of the duration of human-megafauna coexistence per region on the specific timing of megafauna extirpation. Thus, our results support the idea that human arrival in south-eastern Australia was an additional stressor to climate perturbations, rather than humans being a ‘super predator’^4^. Indeed, human populations were most likely small at the time of arrival due to low growth rates that are consistent with high mobility^25^ compared to the period when human population growth increased following the Last Glacial Maximum (~23–19 ka) and the mid-Holocene climatic optimum (~7–5 ka)^26,27^.

An alternative hypothesis is that human populations were kept small by the landscape’s low carrying capacity typically experienced by species expanding into new environments^28,29^, as well as the rapid loss of a main food source following the demise of megafauna species^30^ in the regions with the shortest windows of temporal coexistence. However, we found that low carrying capacity was mostly climate-driven due to lower mean annual precipitation and freshwater availability (see Supplementary Figure 6); this, along with high mean annual temperature, led to both lower net primary production and harsher desert conditions than during the Last Glacial Maximum (see Supplementary Fig. 7a′–e′). We argue that as for many other species^31^, human population density likely followed a slow growth pattern, quickly reaching a density plateau after establishing in the new environment due to competition with other species occupying the same landscape.

This alternative scenario of extinction is even more relevant in areas where climate was the only plausible driver of megafauna extinctions—in areas where there was an absence of temporal human-megafauna coexistence such as in Tasmania (Fig. 2a, blue areas, and ref. ^32^) because mean annual precipitation, mean freshwater availability, and mean annual desert fraction best explained the timing of megafauna extinctions there (Table 1 and Fig. 3b). In addition to local drought conditions, the pattern of primary production best explained the pattern of megafauna extirpations (Table 1, Fig. 3c and Supplementary Fig. 6d) during the prevailing cold conditions (i.e., up to 4° C lower than the conditions dominating during the Last Glacial Maximum; Supplementary Fig. 7) and low atmospheric CO_2_ concentrations that would have limited plant growth^33^ at that time.

We acknowledge that our approach has some potential limitations, such as disregarding the possible feedbacks of human-induced fire^34^ and the complexity of interspecific interactions at the community level^35^. Despite some available data describing fire regimes in Australia^36^, our approach cannot distinguish human- from climate-driven fire patterns. Further, the low spatial coverage of high-quality megafauna fossil data in Australia still makes it impossible to generate outputs at the species level. However, by adding a spatial component (i.e, the bearing) we demonstrated a combined effect of climate and humans on the regional patterns of megafauna extirpations. In addition, we found that a shift in net primary production was the main driver of the regional pattern of megafauna extirpations in areas where humans were absent.

Our results therefore (i) highlight the limitations of standard (i.e., assuming no explicit spatial connectivity between sites) chronological analyses that are inadequate for describing the multidimentional complexity of megafauna extirpations in Australia. Our work also revealed that (ii) climatic effects on ecosystem resources are more complex than a simple interpretation that rainfall instead of temperature was the main climatic variable driving extirpations in Australia^37,38^. Here, freshwater availability essential to plant and animal life in an arid continent is the result of intricate physical processes between rainfall, evapotranspiration and geology. Finally, our work (iii) challenges the notion built from simple chronological analyses that anatomically modern humans were the principal drivers of extinctions^1,6,10^ in Australia, and that climate variation was at best a secondary contributor^9^. Instead, we have revealed at least for south-eastern Australia a more complex scenario where climate change could have limited the amount of available resources for species, but that human appearance was likely another important and necessary contributor to explain megafauna extirpations in many parts of the landscape.

## Methods

### Overview of the modelling approach

The primary data required to build chronological analyses are derived from dates of fossilised remains (or dated human artefacts expressing the timing of first human occurrence)^37,38^. Because of sporadic fossilisation processes, the youngest dated fossil record in a time series is unlikely in the extreme to reflect the true timing of a species’ demise, just as much as the oldest dated fossil is unlikely to indicate the true timing of a species’ arrival (i.e., the Signor-Lipps effect)^13^. A whole suite of statistical models can be applied to correct for this and estimate an unbiased timing of arrival or extinction (see ref. ^14^), but the lack of spatial resolution from biased sampling arising from a restricted spatial distribution of fossil sites constrains the inferences of extinction timing either to specific locations or to broad-scale (e.g., continental), spatially averaged phenomena^39–42^.

To create continuous maps of unbiased timings of megafauna extirpation and human arrival given the inherent rarity of fossil sites, and to investigate the geographic patterns of the timing of regional extinctions, we designed and implemented a new statistical approach to infer spatial patterns of the regional timing of megafauna extirpations and initial human appearance across south-eastern Australia. Our approach explains these dynamics as a function of the spatial patterns of palaeoclimate change simulated over the last 120,000 years. We successfully validated the approach against hundreds of scenarios of simulated datasets (Supplementary Fig. 2), and the error map presents a confidence interval of the duration of the window of coexistence/non-coexistence of <3000 years for most of the study area; this indicates robust extrapolation performance of the method based on few data (Supplementary Fig. 3). We restricted our study to south-eastern Australia (Fig. 1a) owing to the scarce and patchy distribution of high-quality data available for other parts of the continent (see ‘Megafauna and human datasets’ below).

### Climate variables

We used a climate reconstruction based on the three-dimensional Earth-system LOVECLIM model of intermediate complexity^19,39^. The model includes representations of the atmosphere, ocean and sea ice, land surface (including a vegetation model that simulates the dynamics of two main terrestrial plant functional types [trees and grasses], as well as deserts), ice sheets, and the carbon cycle. We simulated 1000-year average climates over the past 120,000 years using LOVECLIM. The original spatial resolution of LOVECLIM is 5.625 × 5.625°, but we downscaled the output resolution to 1 × 1° using bilinear interpolation because it retains the integrity and limitations of the original model output, where orography is highly smoothed relative to the real world^40^. For each grid cell and each temporal snapshot, we extracted mean annual temperature, mean annual precipitation, mean annual available freshwater (calculated as the difference between the mean annual evapotranspiration and mean annual precipitation), mean annual desert fraction, and mean annual net primary production. We then calculated the anomaly of each of these variables by substracting their value every 1000 years for the last 120,000 years from their respective average values calculated over the interval 50–30 ka (i.e., time window of the main megafauna extinction events in Australia^7^) to capture the temporal variation in climate during the period of megafauna extinctions. These five climate variables characterise both natural resources for megafauna species^41^ and constraints to human appearance and movement^23,39^.

### Megafauna and human datasets

From the 2138 records of extinct fauna species in the *FosSahul* database^42^, we quality-rated each fossil date following the A* to C scale (from ‘high quality’ to ‘unreliable’) based on objective criteria^43^, including reliability in sample pretreatment and measurement (see Supplementary Table 1). We only used A* and A quality-rated dates (Fig. 1) and further excluded all data for which ages were obtained from materials in depositional context below or above the fossil(s) of interest. We used the same methodology to extract data from the 6349 archaeological records (ref. ^44^, Supplementary Table 1). There are few human skeletal remains in the database; thus, human presence is largely restricted to artefacts related to human activities (for example, hearth charcoal, shell middens, stone tools and rock art). We excluded an age estimate of 62 ± 6 ka from Lake Mungo, because it is highly contested^45^.

### Estimating the timing of extirpation and initial arrival

We applied a maximum-likelihood method to correct for the Signor-Lipps effect first developed by Solow^46^ that we adapted for spatial inference of both megafauna extirpation and human appearance patterns. The original (non-spatial) version of the model assumes that the true ages (e.g., estimated using radiocarbon techniques) of specimens within a time series, *T*_1_,…, *T*_*n*_, are independent and uniformly distributed over the interval (*T*_ext_, *γ*), which correspond to the extirpation (*T*_ext_) and settlement time (*γ*) for the taxon under investigation, respectively. In contrast to the original version of this model that assumes a constant dating error across samples, we assumed that the radiometric errors associated with each estimated age were approximately normally distributed^47^. Thus, $\widehat T_k$ estimates the *k*^th^ age assuming the true age *T*_*k*_ follows:

1 $$\widehat T_k\sim g\left( {T_k,\sigma _{\left( k \right)}^2} \right)$$

with *g* being the Gaussian probability density function describing the radiometric error *σ*. The estimated timing of megafauna extinction $\widehat T_{{\mathrm{ext}}}$ and the estimated timing of initial human arrival $\widehat \gamma$ are then calculated by numerically maximising the log-likelihood ℒ (*ϑ*) over *ϑ*:

2 $${\cal{L}}\left( \vartheta \right) = \sum_{k = 1}^{n} {\log h\left( {\widehat T_{k}} \right)}$$

with *ϑ* representing either $\widehat T_{{\mathrm{ext}}}$ or $\widehat \gamma$ and *h* the probability density of $\widehat T_k$

3 $$h\left( {\widehat T_k} \right) = \int_\gamma ^{T_{{\mathrm{ext}}}} {g\left( {t,\,\sigma _k^2} \right)\frac{{dt}}{{T_{{\mathrm{ext}}} - \gamma }}}$$

To adapt this approach to questions of spatial inference, let us define *W* as the spatial landscape, and *T*_*ϑ*(*x*)_ as either the date of megafauna extirpation or initial human arrival at a given grid cell *x* in *W*. We gridded *W* at a spatial resolution of 1 × 1° (i.e., the same resolution as the climate data for consistency), and then used Eq. (3) to estimate *T*_*ϑ*(*x*)_ in each grid cell of *W*. However, to estimate *T*_*ϑ*(*x*)_ in each grid cell, even for those cells without data, we used the entire dataset of *W* and attributed a weight for each age on *T*_*ϑ*(*x*)_ as a function of the distance of the age relative to *x*. Thus, the closer the age to *x*, the more weight this age will have on *T*_*ϑ*(*x*)_. Calculating the distance of each age relative to a given grid cell *x* in *W* is similar to calculating a local density of age around *x*.

First, we assumed that ($\widehat T_1$,…, $\widehat T_n$) are individual point estimates of the taxon’s presence (i.e., megafauna or human) based on independently discovered dated specimens found at location sites (*x*_1_,…, *x*_*N*_). According to Eq. (1), the estimated timing of human appearance or megafauna extirpation $\widehat T_\vartheta \left( x \right)$ in a grid cell *x* is calculated by numerically maximising ℒ_*x*_(*T*_*ϑ*_) over *ϑ* across *W*:

4 $${\cal{L}}_{x}\left( {T_{\vartheta} } \right) = \sum_{k = 1}^{n} {\log h_{\gamma} \left( {\widehat T_{k}} \right)^{w\left( {x - x_{k}} \right)}}$$

where *w*(*x*−*x*_*k*_) is a weighting factor, such that Σ*w*(*x*−*x*_*k*_) = 1. The standard procedure in estimating local density is to select a weighting factor proportional to:

5 $$w\left( z \right) = \frac{1}{b} \times g_0\left( {\frac{z}{b}} \right)$$

where *g*_0_ = the density of the standardised Gaussian distribution and *b* is an optimised bandwidth (see details hereafter) so that the larger the bandwidth, the more dated neighbour specimens from *x* over *W* are considered in the approximation of the distribution^48,49^.

Including a spatial component such as estimating local density generates a spatial bias in $\widehat T_\vartheta \left( x \right)$^48^ that depends on the size of the bandwidth *b*: wider *b* take specimens farther away from *x* into account in *w*(*z*), adding some bias into $\widehat T_\vartheta \left( x \right)$, whereas narrower *b* take fewer specimens into account in *w*(*z*), thus increasing the variance of $\widehat T_\vartheta \left( x \right)$. Here, the idea is to find an optimal size for *b* to obtain the lowest values of both bias and variance into $\widehat T_\vartheta \left( x \right)$. We corrected for this bias by using a simulation-based spatial bias-correction procedure (Supplementary Fig. 1). This procedure estimates the bias generated by the model at each grid cell (along with its variance across space) given an optimised bandwidth size. The first step (model inference, see Supplementary Fig. 1 step 1) calculates a ‘preliminary’ spatial estimate from the model for each grid cell for a predefined bandwidth size (arbitrarily set to one tenth the maximum pairwise distance between the data at each location).

In the following steps we assumed this ‘preliminary’ estimate to be a true date of extinction or arrival (*T*_*ϑ*(*p*)_) for each grid cell. Based on these *T*_*ϑ*(*p*)_, we generated *n* simulated time series (*n* = 100, Supplementary Fig. 1 step 2) following the same spatial pattern and characteristics (i.e., number of ages, laboratory dating error; see details in ref. ^18^) as the dated record. We then inferred for each of the *n* simulation time series a spatial estimate of timing of extinction per grid cell using the model (Supplementary Fig. 1 step 3), and we calculated the average estimate for each grid cell across the *n* simulation time series (Supplementary Fig. 1 step 4). By comparing the average estimate with *T*_*ϑ*(*p*)_, we calculated a mean bias (i.e., $B_{({{\widehat{T}}_{\vartheta}} ({x}))}$) in each grid cell *x* and the associated variance (i.e., ${{\sigma}^{2}}_{ {{\widehat{T}}_{\vartheta}} ({x})}$) across space that are used to approximate the integrated mean-squared error across the landscape *W* (*E*):

6 $$E \simeq \sum_{x} \left( {{\sigma ^{2}}}_{{\widehat{T}}_\vartheta (x)} + {B_{(\widehat T_\vartheta(x))}}^{2} \right)$$

We then repeated steps 1 to 4 (Supplementary Fig. 1) using different bandwidth sizes (assuming that the size of the bandwidth is the same for each grid cell) until we obtained the lowest *E* (Supplementary Fig. 1 steps 5 and 6). In the final step, we subtracted this bias spatially from *T*_*ϑ*(*p*)_ in each grid cell to provide a final and spatially unbiased estimate of extinction timing (Supplementary Fig. 1 step 7) given the optimal bandwidth.

### Bearing of the spatial gradients

We calculated spatial gradients of (i) the timing of megafauna extirpation, (ii) timing of initial human arrival, and (iii) the five climate-driven variable generated by LOVECLIM from a 3 × 3 grid-cell neighbourhood using the average maximum technique modified to accommodate different cell widths at different latitudes. Following ref. ^50^, we calculated the average ‘north-south’ and ‘west-east’ gradients for the focal cell, excluding any missing values (usually along coastlines) using weightings of 1 and 2 for cells diagonal and adjacent, respectively, to the focal cell^51^. We calculated spatial gradients as the vector sum of the ‘north-south’ and ‘west-east’ gradients, with the associated vector angle giving the bearing of the gradient^50^.

### Explanatory factor analyses

We constructed and compared generalised least-squares models to determine which predictor among the climate variables (see details in ‘Climate variables’ above) and initial human appearance best described spatial variation in the timing and bearing of megafauna extirpations. Generalised least-squares models have the advantage of accounting for spatial autocorrelation in predictor variables (i.e., model residuals are correlated and each observation does not contribute a full degree of freedom)^52^. Because of the large number of possible combinations of predictor variables (including their interactions), we used a phasic approach. We first computed and compared the Akaike’s information criterion weights (corrected for small sample size: *w*AIC_*c*_)^53^ of 29 and 60 generalised least-squares models assuming a Gaussian spatial autocorrelation structure, in areas with no coexistence between humans and megafauna (29 models) and areas with coexistence (60 models), respectively—these correspond to all combinations of predictor variables excluding their interactions. We took climate values from LOVECLIM^19,39^ for the time period in each grid cell corresponding to the estimated timing of megafauna extirpation and its confidence interval.

We selected the top-ranked model as the model with the highest *w*AIC_*c*_ and tested this model for the effect of all possible interactions among predictor variables. We computed *w*AIC_*c*_ for these newly constructed models and ranked them against the top-ranked model without interactions to select the final top-ranked model. From this final model, we evaluated the relative importance of each predictor variable by calculating the change in likelihood (i.e., a metric for the model’s goodness of fit) when this predictor is left out of the full model. This procedure is equivalent to a sensitivity analysis of the final model to each of its predictors to quantify and rank the relative importance of each predictor in the full model. We also accounted for a possible temporal lag between climate conditions and megafauna extirpation. Here, we calculated the temporal lag by regressing extirpation against climate from 1000 to 5000 years (at a 1000-year time step = 5 temporal-lag scenarios) for the period earlier than the estimated timing of megafauna extirpation in each grid cell (Fig. 1b). For each new time-lag scenario, we reconstructed and compared a new set of generalised least-squares models to determine which predictor among these best described spatial variation in the timing and bearing of megafauna extirpations.

## Supplementary information

Supplementary Information

Description of Additional Supplementary Files

Supplementary Data 1

Supplementary Data 2

## Data Availability

The data supporting the Fig. 1 of this study are available within the paper as Supplementary Table 1. All data generated and analysed during this study as well as the raw data values underlying all reported averages in graphs and charts (i.e., Fig. 3 and Supplementary Figs. 4 and 5) are available at github.com/FredSaltre/SEOZ_megafauna_extirpation. The authors declare that the source data underlying Figs. 1a, 2a–d, 6d, h and 7c and Supplementary Figs. 1a and 5d are provided as a Source Data file.

## Source Data

Source Data

## Code availability

The code is freely available on github.com/FredSaltre/SEOZ_megafauna_extirpation.
