# Supplementary material for: Climate-human interaction associated with southeast Australian megafauna extinction patterns
Source: Nat Commun. 2019 Nov 22;10:5311. doi: 10.1038/s41467-019-13277-0 (PMC6876570; doi:10.1038/s41467-019-13277-0)
Supplement: Supplementary file 1 — Supplementary Information [file 41467_2019_13277_MOESM1_ESM.pdf]

Supplementary Information

Climate-human interaction associated with southeast Australian megafauna-extinction patterns

Saltré et al.

Supplementary Figures

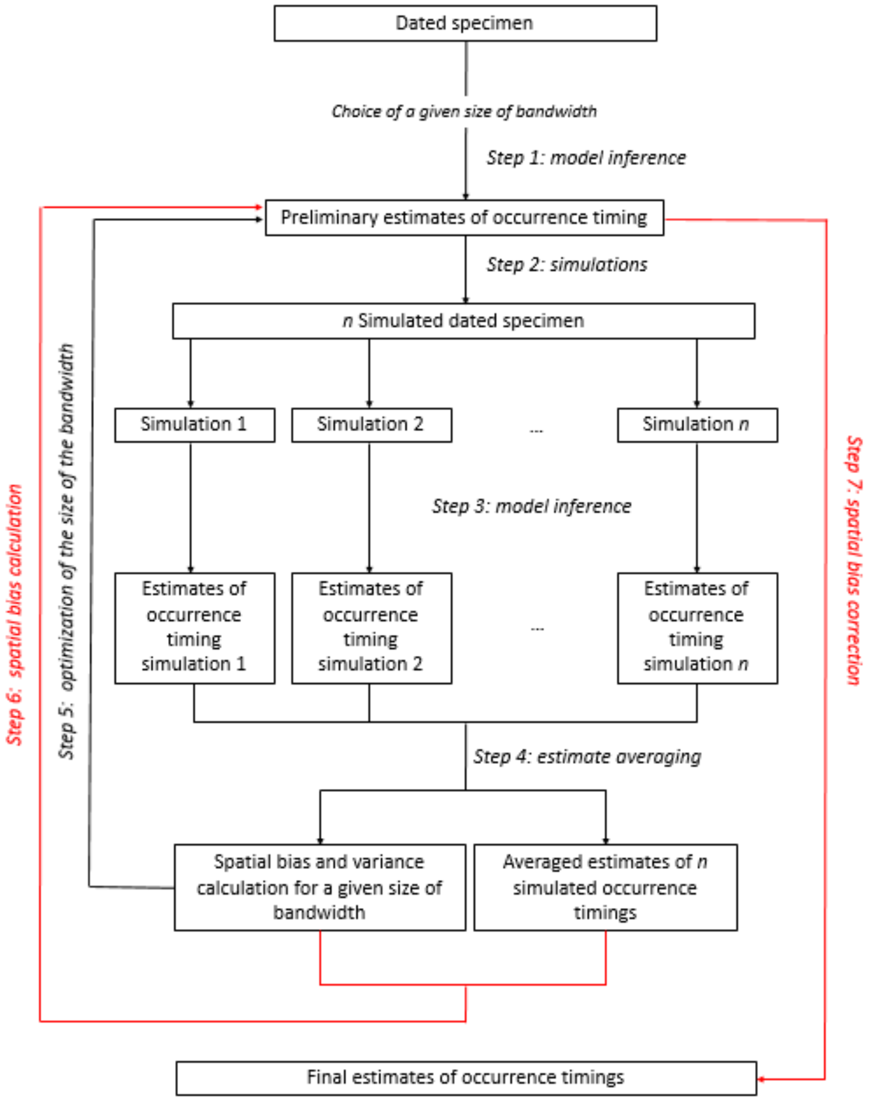

Supplementary Figure 1 — Flow chart of the spatial bias-correction procedure.

Supplementary Note 1: Model validation and confidence intervals — regional estimates of first occurrence and extirpation

We evaluated the ability of our statistical approach to reproduce the regional estimates of simulated (i.e., already known) timings of first occurrence and extirpation. First, we generated simulated maps of timings of initial human occurrence and megafauna extirpation following two main scenarios: (1) the first scenario describes a gradual initial appearance (or gradual extirpation) across space (Supplementary Figure 2, lower-left panel), whereas the second scenario describes two entrances of appearance/extirpation in the landscape (Supplementary Figure 2, bottom-right panel). We called these maps 'benchmark maps'. Next, for each timing of appearance / extirpation we simulated time-series ages assuming a uniform distribution, along with their associated standard deviations, such that older ages have a larger standard deviation. These time series represent either dated fossil or archaeological specimens. Thus, we applied our statistical approach to these simulated fossil / archaeological specimens to infer a 'new map' of regional timing of first arrival or extirpation. We compared this new map to the original benchmark map. We repeated the entire process one hundred times to account for potential variability in our results.

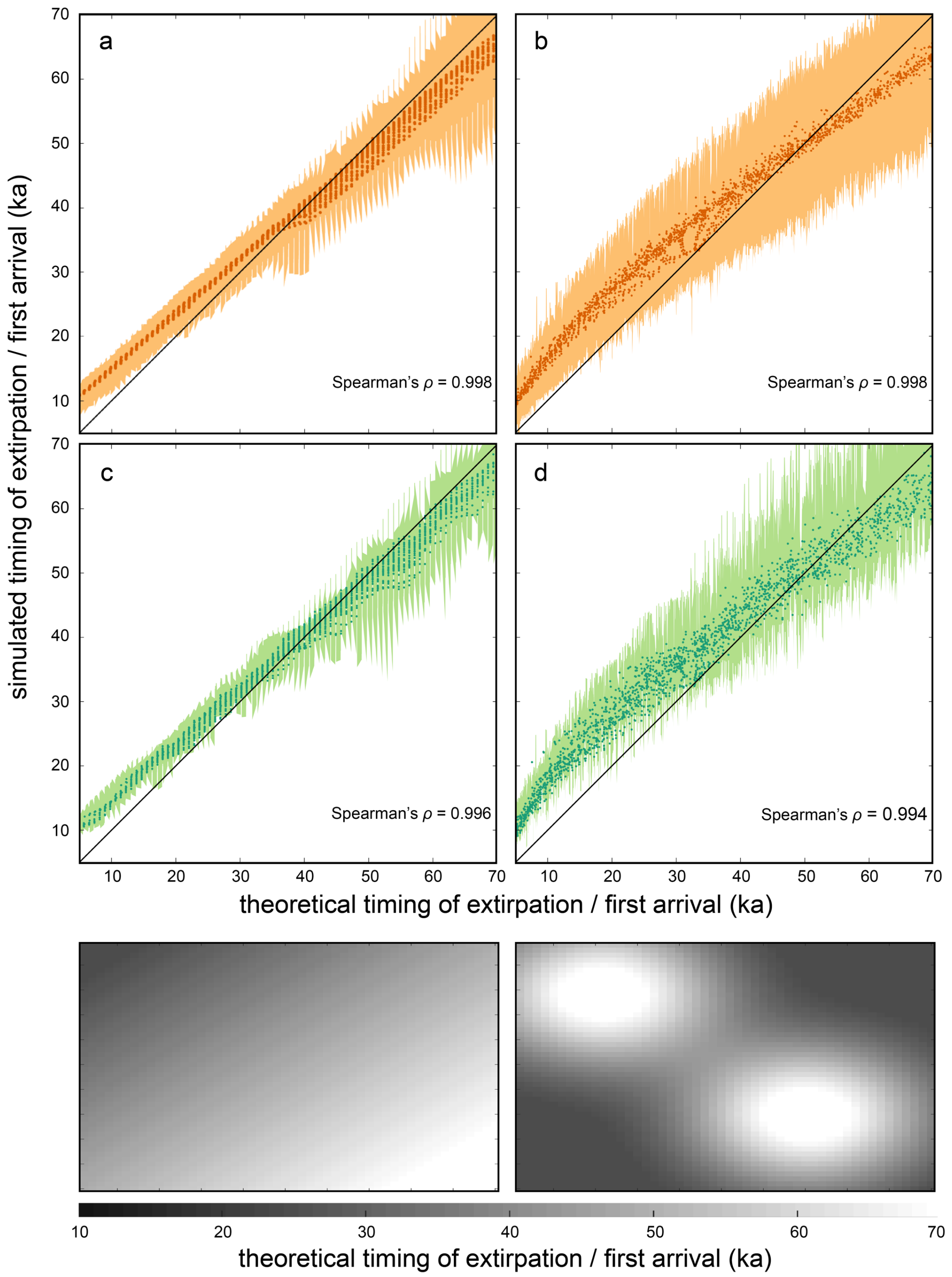

Supplementary Figure 2 — Pairwise comparison between observed and estimated regional timing of extirpation (**a**, **b**) and regional timing of first arrival (**c**, **d**) for scenarios based on (**a, c**) gradual appearance / extirpation from a single entrance in the landscape and (**b, d**) two entrances of appearance / extirpation in the landscape. The scatterplot indicates the timing of first arrival or extirpation for each grid cell of the 100 simulated maps (*y*-axis) against the timing of first arrival or extirpation for each grid cell of the original benchmark map (*x*-axis). Correlations (Spearman's *ρ* rank correlation coefficient) are calculated over 100 replicates of the pairwise comparisons of the theoretical and inferred timing of first arrival / extirpation. Bold black lines indicate the median correlation across all repetitions, dark-shaded envelopes represent limits determined by the 25th and 75th

percentiles, and light-shaded envelopes indicate the lower and upper limits determined by the 2.5th and 97.5th percentiles. The grey dotted line in each panel assumes a perfect relationship between observed and inferred values. Bottom panels display the type of appearance pattern for each scenario (left = single entrance; right = two entrances).

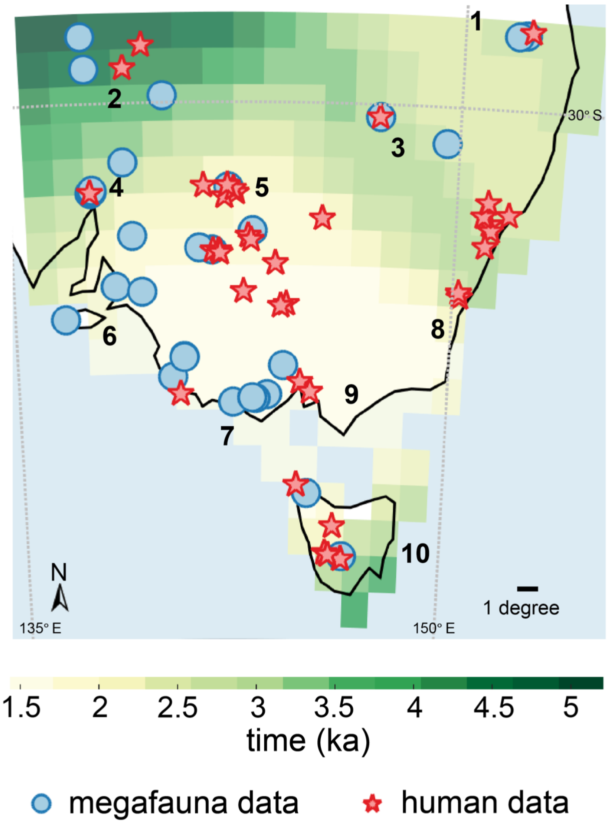

Supplementary Figure 3 — Confidence interval of the duration of the window of coexistence / non-coexistence between humans and megafauna. We calculated the confidence interval for each grid cell as the difference between the 75th and the 25th percentile of the distribution generated by substracting from 10,000 resampled estimated timings of extirpation the 10,000 resampled estimated timings of initial human arrival. These resampled estimated timings (i.e., megafauna extirpation and initial human arrival) are determine by random resampling withing the estimated confidence interval returned by the model inferring the timing of megafauna extinction **(T_{ext})**, and initial human arrival **(γ)**, respectively (see detail of the model in Methods).

Supplementary Note 2: Sensitivity of the size of the area of coexistence and non-coexistance to the approach used to account for the confidence intervals around regional model estimates

In Figure 2, we calculated the areas of coexistence (brown cells) and non-coexistence (blue cells) between humans and megafauna. We accounted for confidence intervals around estimated timings so that 'coexistence' indicates when the lower confidence limit of the timing of initial human arrival is older than the lower confidence interval limit of megafauna extirpation. However, a comparison of lower confidence intervals is 'statistically liberal', from which false positives can potentially arise in some occasions. We evaluated the robustness of our approach by calculating the areas of coexistence and non-coexistence using (*i*) a 'statistical standard criterion' (i.e., mapping the grid cells in which the lower confidence limit of humans is older than the mean age of megafauna extirpation; Supplementary Figure 4) and (*ii*) a more 'statistically conservative criterion' (i.e., mapping grid cells in which the lower limit of human arrival is older than the upper limit of megafauna extirpation; Supplementary Figure 5).

Mapping the areas of coexistence and non-coexistance with these new criteria increased the area of non-coexistence by < 20% compared to using the statistically liberal criterion mostly in locations 6 (Kangaroo Island), 7 (southeast coastal plains), and 9 (southern coast of mainland Australia; Figure 1). Ignoring the criteria we used to calculate both coexistence and non-coexistence areas (i.e., liberal, standard, or conservative), none of the generalized least-squares models we tested explained any variation in the timing alone of megafauna extirpation in areas of human-megafauna coexistence (i.e., ~ 0% of variance explained), and the top-ranked model explaining variation in the spatial pattern of timing of extirpation (bearing = Ext_b; Table 1) in non-coexistence areas was driven mainly by the pattern of changes in mean annual net primary production (Fig. 3c, Supplementary Figure 3c and Supplementary Figure 4c). The spatial pattern of timing of extirpation (bearing = Ext_b; Table 1) in coexistence areas is still best explained by a combination of the bearing of the

timing of human arrival (H_b), and the bearing of climate variables. Both liberal and standard criteria indicate that the bearing of the timing of human arrival (H_b) and the bearing of water availability (EminP_b) had the strongest effects on the spatial pattern of timing of extirpation, whereas a more conservative approach supported some effect of the bearing of the timing of human arrival (H_b) and the bearing of the changes in food resources (i.e., net primary production, NPP_b) driven by an interaction between temperature (T_b) and precipitation (P_b).

A generalized least-squares model built on mean annual precipitation anomaly (P), mean annual water availability anomaly (EminP), the percentage of desert fraction (DF), and their respective interactions (i.e., $P \times \mathrm{EminP}$ and $\mathrm{EminP} \times \mathrm{DF}$) as predictive variables provided the highest support to describe the timing of extirpation in these areas whatever the criteria we used to defined the non-coexistence areas. However, the addition of the extra area of non-coexistence in location 1 (i.e., Sandy Hollow Creek; Fig. 1) by using the standard criterion show another regional effect including mean annual temperature and mean annual net primary production.

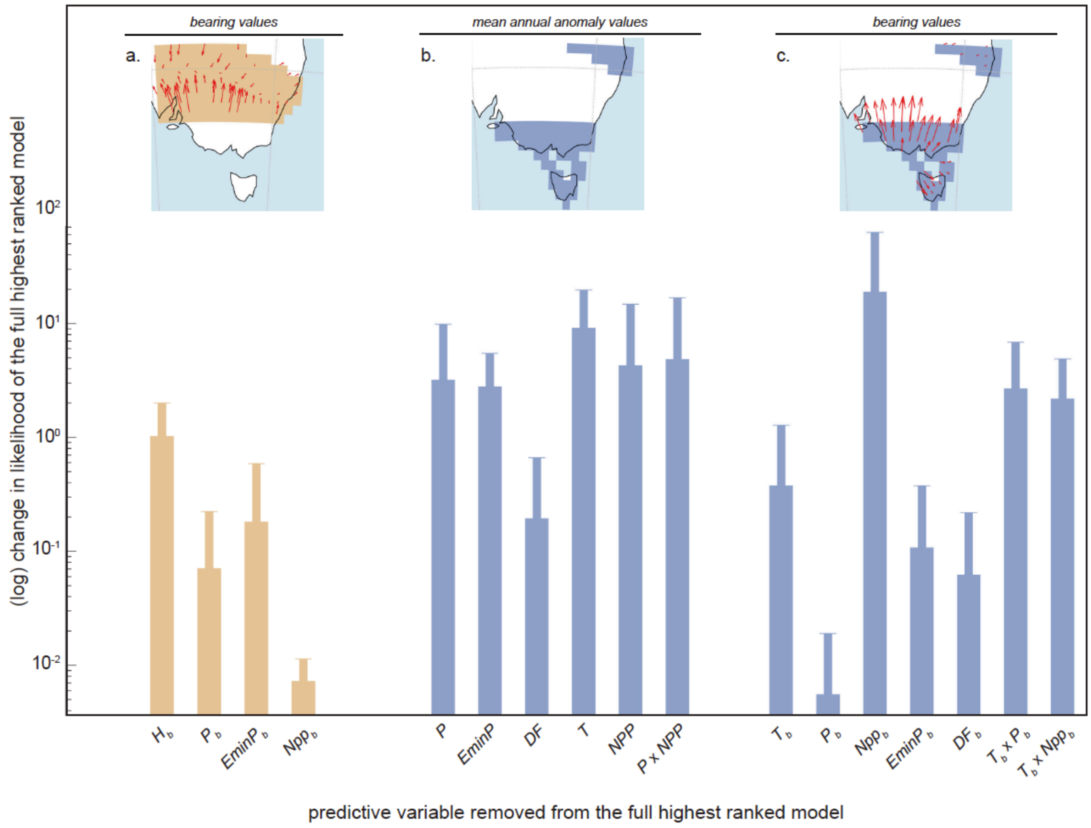

Supplementary Figure 4 — Relative importance of predictor variables for the top-ranked generalized least-squares model assuming a Gaussian spatial autocorrelation structure best decribing (**a**) the spatial gradient (bearings) of megafauna extirpation (Ext_b) in human-megafauna coexistence areas, (**b**) the timing of megafauna extirpation (Ext_t) in human-megafauna non-coexistence areas, and (**c**) the bearings of megafauna extirpation (Ext_b) in human-megafauna non-coexistence areas. We calculated the human-megafauna coexistence areas using a 'statistical standard' approach criterion by mapping the grid cells in which the lower confidence limit of humans is older than the mean age of megafauna extirpation. These areas of coexistence and non-coexistence are displayed in the top panel of each barplot along with red arrows indicating the bearing of megafauna extinction patterns. The relative importance of each predictor variable is calculated as the change in the full model likelihood when one of its predictor variables is removed. Climate predictors were from LOVECLIM[1,2] for the time period in each grid cell corresponding to the estimated timing of megafauna extirpation and its confidence interval. Predictor variables of the model describing Ext_t are mean annual temperature anomaly (***T***), mean annual precipitation anomaly (***P***), mean annual freshwater availability anomaly (**EminP**), mean annual net primary production anomaly (**NPP**), and the fraction of desert anomaly within the grid cell (**DF**). Climate anomalies are calculated relative to the 50-30 ka mean time period (see Methods). Predictor variables subscripted *b* ($\boldsymbol{T_b}$, $\mathbf{EminP}_b$) indicate that we used the directional bearing of these climate variables, including the directional vectors for the timing of initial human arrival ($\boldsymbol{H_b}$), to build the model describing the spatial pattern bearing of megafauna extirpation ($\mathbf{Ext}_b$). For clarity, we did not present the results describing Ext_t for the areas with human and megafauna coexistence because we did not have any relevant model (i.e., percentage of variance explained by the models ~ 0%) in those areas. For each variable, error bars represent the standard deviation of the relative importance of predictor variables for the top-ranked generalized least-squares model accounting for the temporal lag by regressing extirpation against climate from 0 to 5000 years (at a 1000-year time step = 5 temporal-lag scenarios, i.e., 6 values of relative importance in total) per grid cell. Source data are provided as a Source Data file.

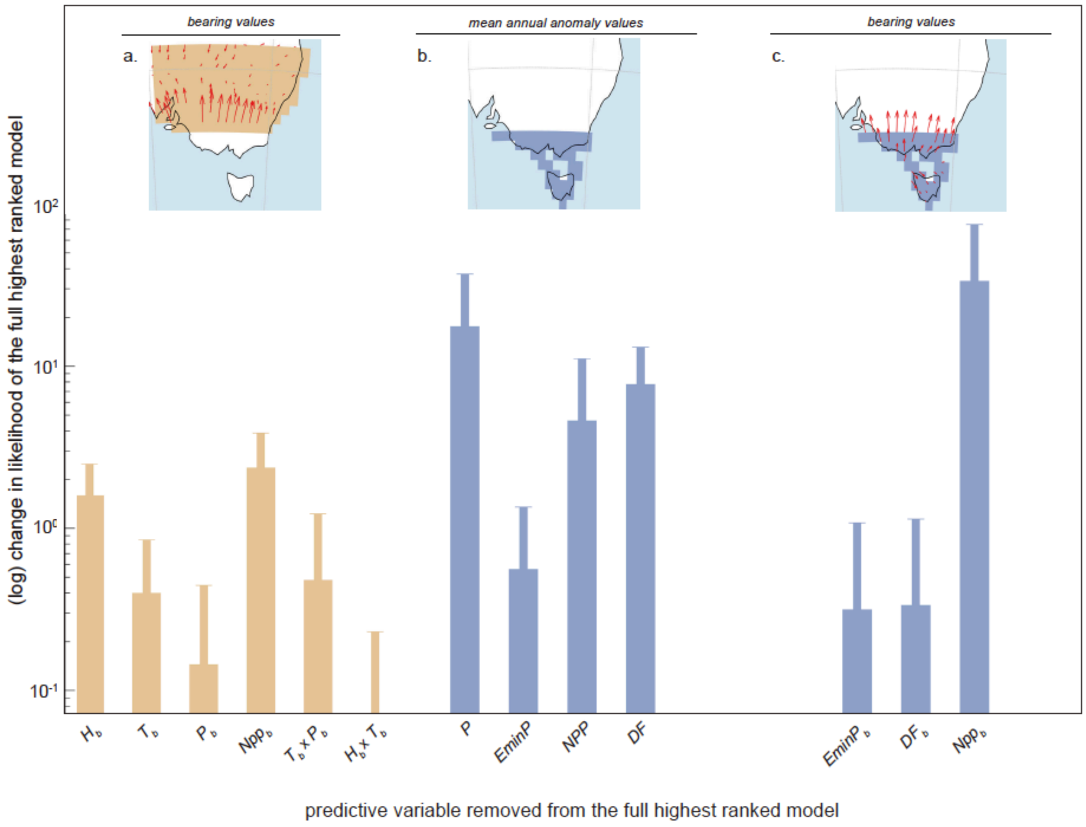

Supplementary Figure 5 — Relative importance of predictor variables for the top-ranked generalized least-squares model assuming a Gaussian spatial autocorrelation structure best decribing (**a**) the spatial gradient (bearings) of megafauna extirpation (Ext_b) in human-megafauna coexistence areas, (**b**) the timing of megafauna extirpation (Ext_t) in human-megafauna non-coexistence areas and (**c**) the bearings of megafauna extirpation (Ext_b) in human-megafauna non-coexistence areas. We calculated the human-megafauna coexistence areas using a 'statistical conservative' approach by mapping the grid cells the lower limit of human arrival is older than the upper limit of megafauna extirpation. These areas of coexistence and non-coexistence are displayed in the top panel of each barplot along with red arrows indicating the bearing of megafauna extinction patterns. The relative importance of each predictor variable is calculated as the change in the full model likelihood when one of its predictor variables is removed. Climate predictors were from LOVECLIM[1,2] for the time period in each grid cell corresponding to the estimated timing of megafauna extirpation and its confidence interval. Predictor variables of the model describing Ext_t are mean annual temperature anomaly (***T***), mean annual precipitation anomaly (***P***), mean annual freshwater availability anomaly (**EminP**), mean annual net primary production anomaly (**NPP**), and the fraction of desert anomaly within the grid cell (**DF**). Climate anomalies are calculated relative to the 50-30 ka mean time period (see Methods). Predictor variables subscripted *b* ($\boldsymbol{T_b}$, $\mathbf{EminP}_b$) indicate that we used the directional bearing of these climate variables, including the directional vectors for the timing of initial human arrival ($\boldsymbol{H_b}$), to build the model describing the spatial pattern bearing of megafauna extirpation ($\mathbf{Ext}_b$). For clarity, we did not present the results describing Ext_t for the areas with human and megafauna coexistence because we

did not have any relevant model (i.e., percentage of variance explained by the models ~ 0%) in those areas. For each variable, error bars represent the standard deviation of the relative importance of predictor variables for the top-ranked generalized least-squares model accounting for the temporal lag by regressing extirpation against climate from 0 to 5000 years (at a 1000-year time step = 5 temporal-lag scenarios, i.e., 6 values of relative importance in total) per grid cell. Source data are provided as a Source Data file.

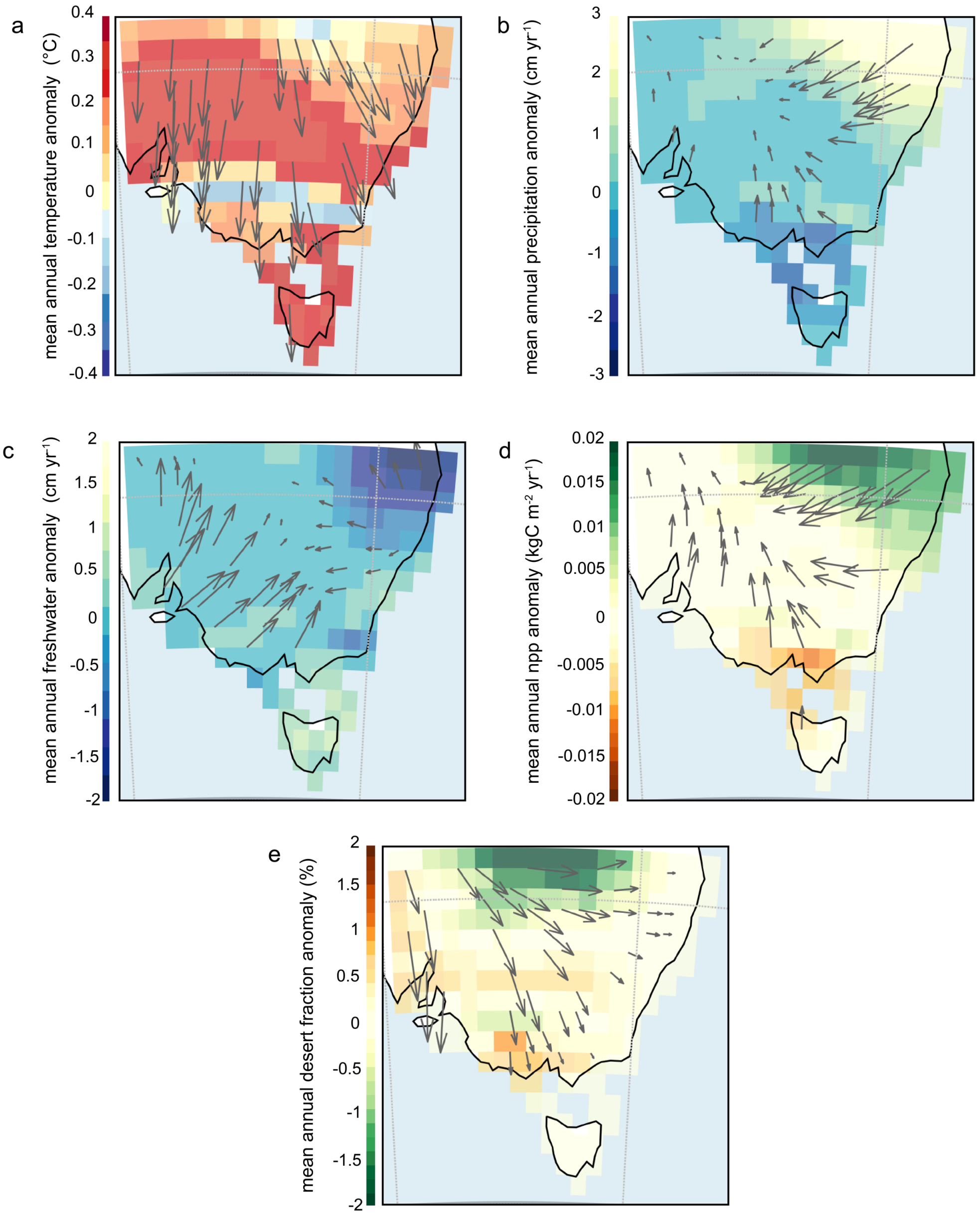

Supplementary Figure 6 — Reconstructed climate anomalies (colour gradients) and spatial gradients (grey arrows) from the LOVECLIM three-dimensional Earth-system model at the time of megafauna extirpation (estimated in Fig. 1) for (a) mean annual temperature anomaly, (b) mean annual precipitation anomaly, (c) freshwater availability anomaly (i.e., evapotranspiration minus precipitation), (d) mean annual net primary production anomaly, and (e) mean annual desert fraction anomaly per grid cell at a spatial resolution of 1 × 1°. Climate anomalies are calculated relative to the 50-30 ka mean time period (see Methods). The spatial gradient of each variable is represented from the highest (i.e., more positive) to the lowest (more negative) value. Grey arrows are not meant to follow the gradient of colour because spatial gradients are calculated from the total climate values (not the anomalies; see Methods). The freshwater climate variable is the difference between evapotransipration minus precipitation so that positive anomalies indicate higher evapotranspiration than precipitation (i.e, less available freshwater).

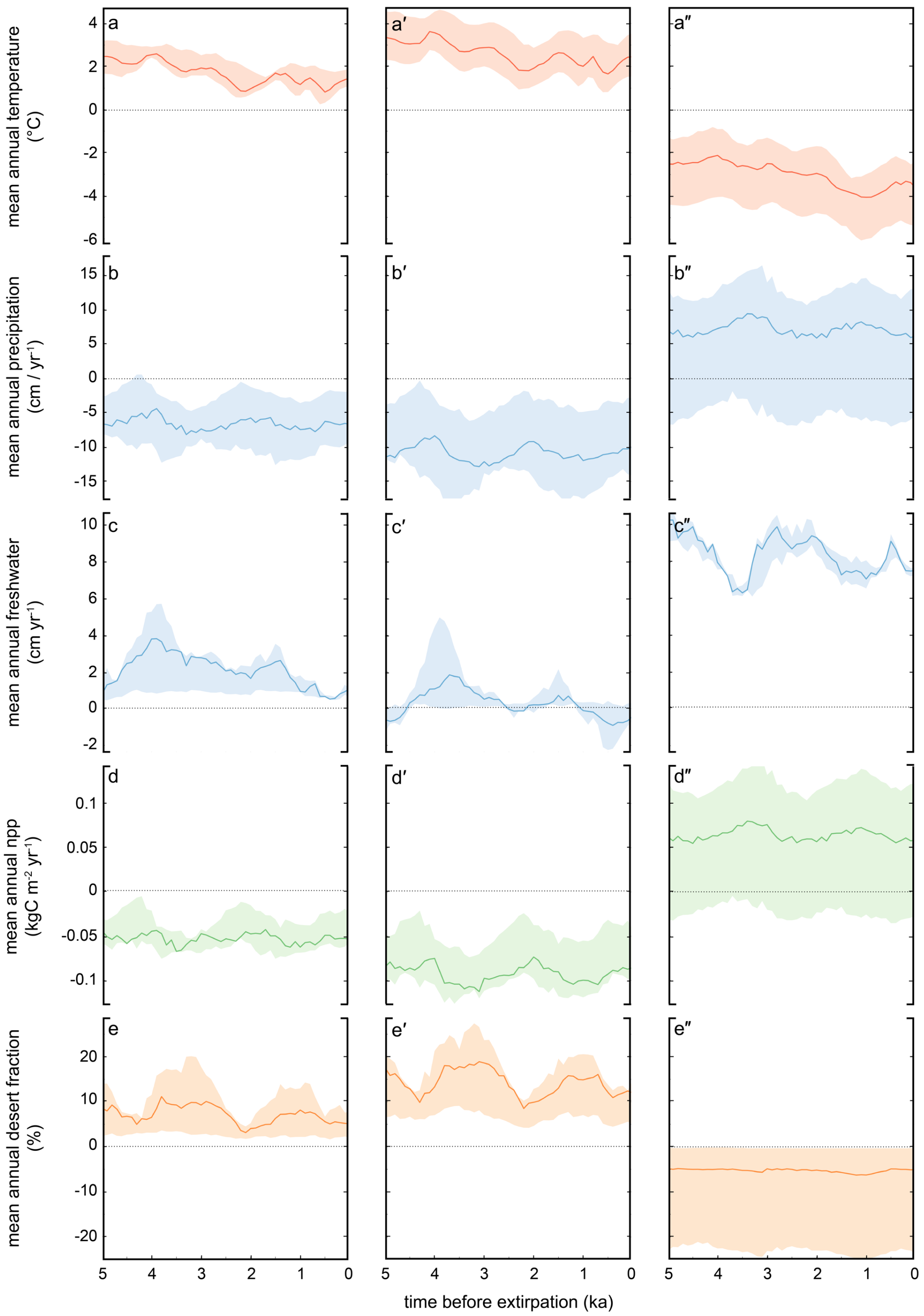

Supplementary Figure 7 — Reconstructed climate anomalies from the LOVECLIM three-dimensional Earth-system model before the estimated timing of megafauna extirpation (estimated in Fig. 1) for (**a, a′ and a″**) mean annual temperature, (**b, b′ and b″**) mean annual precipitation, (**c, c′ and c″**) freshwater availability (i.e., evapotranspiration minus precipitation), (**d, d′ and d″**) mean annual net primary production, and (**e, e′ and e″**) mean annual desert fraction per grid cell at a spatial resolution of 1 × 1°, and for (**a-e**) the entire study area (see Fig. 1a), (**a′-e′**) human-megafauna coexistence areas (Fig. 2a, brown area), and (**a″-e″**) human-megafauna non-

coexistance areas (Fig. 2a, brown area). For each grid cell, we reported climate values 0 to 5 ka in 1-ka increments before the timing of extinction. Bold, coloured lines indicate the median values across all grid cells of the area, and shaded envelopes represent limits determined by the 25th and 75th percentiles. Climate anomalies are calculated for each grid cell relative to the Last Glacial Maximum (20-18 ka year mean) represented in each panel by the black dotted line.

Supplementary References

1 Goosse, H. *et al.* Description of the Earth system model of intermediate complexity LOVECLIM version 1.2. *Geosci. Model Dev.* **3**, 603-633, (2010).

2 Timmermann, A. & Friedrich, T. Late Pleistocene climate drivers of early human migration. *Nature* **538**, 92-95, (2016).
