# Supplementary material for: Climate-human interaction associated with southeast Australian megafauna extinction patterns
Source: Nat Commun. 2019 Nov 22;10:5311. doi: 10.1038/s41467-019-13277-0 (PMC6876570; doi:10.1038/s41467-019-13277-0)
Supplement: Supplementary file 2 — Description of Additional Supplementary Files [file 41467_2019_13277_MOESM2_ESM.pdf]

Description of Additional Supplementary Files

File Name: Supplementary Data 1
Description: Fossil records and archaeological evidences extracted from the FosSahul database used to infer regional timings for megafauna extirpation and initial human occurrence.

File Name: Supplementary Data 2
Description: — Generalized least-squares models containing climate (T = mean annual temperature, P = mean annual precipitation, EminP = mean annual freshwater availability, NPP = mean annual net primary production and DF = fraction of desert within the grid cell), and human predictors (H) to describe (i) the timing of megafauna extirpation (Ext(t)) in human-megafauna non-coexistence areas (n/coexist) and (ii) in human-megafauna coexistence areas (coexist), (iii) the bearing of timing of megafauna extirpation (Ext(b)) in human-megafauna non-coexistence areas (n/coexist) and (iv) in areas with coexistence (coexist). For each of these four scenarios we included five temporal lags (Lag) between the climate from 0 to 5 ky (at a 1 ky-year time step, with ky = 1000 years) for the period earlier than the estimated timing of megafauna extirpation in each grid cell. Predictor variables indexed as (a) (i.e., T(a), P(a), Npp(a), EminP(a), DF(a)) indicate that we used the mean annual anomaly relative to the period 50-30 ka for these variables, whereas predictor variables indexed as (b) (i.e., T(b), P(b), Npp(b), EminP(b), DF(b) and H(b)) indicate that we used the directional bearing. Shown are the number of parameters (k), metric of the model's structural goodness of fit (%DE), minimized negative log-likelihood (LogLik), weight scaled to a sum of 1 ($wAIC_c$) and the Akaike's information criterion corrected for small sample sizes (AIC_c) for all models.
